# The Prognostic Value of Integrated Analysis of Inflammation and Hypoxia-Related Genes in Idiopathic Pulmonary Fibrosis

**DOI:** 10.3389/fimmu.2022.730186

**Published:** 2022-03-03

**Authors:** Jun Liu, Liming Gu, Wenli Li

**Affiliations:** ^1^ Reproductive Medicine Center, Yue Bei People’s Hospital, Shantou University Medical College, Shaoguan, China; ^2^ Medical Research Center, Yue Bei People’s Hospital, Shantou University Medical College, Shaoguan, China; ^3^ Department of Microbiology and Immunology, Shantou University Medical College, Shantou, China; ^4^ Guangdong Provincial Key Laboratory of Infectious Diseases and Molecular Immunopathology, Shantou University Medical College, Shantou, China

**Keywords:** ssGSEA, WGCNA, IPF, inflammation, hypoxia

## Abstract

Currently, the aetiology and pathogenesis of idiopathic pulmonary fibrosis (IPF) are still largely unclear. Moreover, patients with IPF exhibit a considerable difference in clinical presentation, treatment, and prognosis. Optimal biomarkers or models for IPF prognosis are lacking. Therefore, this study quantified the levels of various hallmarks using a single-sample gene set enrichment analysis algorithm. The hazard ration was calculated using Univariate Cox regression analysis based on the transcriptomic profile of bronchoalveolar lavage cells and clinical survival information. Afterwards, weighted Gene Co-expression Network Analysis was performed to construct a network between gene expression, inflammation response, and hypoxia. Subsequently, univariate Cox, random forest, and multivariate Cox regressions were applied to develop a robust inflammation and hypoxia-related gene signature for predicting clinical outcomes in patients with IPF. Furthermore, a nomogram was constructed to calculate risk assessment. The inflammation response and hypoxia were identified as latent risk factors for patients with IPF. Five genes, including HS3ST1, WFDC2, SPP1, TFPI, and CDC42EP2, were identified that formed the inflammation-hypoxia-related gene signature. Kaplan-Meier plotter showed that the patients with high-risk scores had a worse prognosis than those with low-risk scores in training and validation cohorts. The time-dependent concordance index and the receiver operating characteristic analysis revealed that the risk model could accurately predict the clinical outcome of patients with IPF. Therefore, this study contributes to elucidating the role of inflammation and hypoxia in IPF, which can aid in assessing individual prognosis and personalised treatment decisions.

## Introduction

Idiopathic pulmonary fibrosis (IPF) is the most common interstitial lung disease of unknown cause, which is characterized by diffused alveolitis and alveolar structure disorder (1). IPF involves the progressive deterioration of lung function, which is characterized by reduced volume of both lungs and thickening of visceral pleura resulting in dyspnea and, eventually, respiratory failure (2–4). The etiology of IPF is still unclear; In addition, there is a lack of specific drugs for treatment (5). Currently, the occurrence of disease is mainly related to alveolar epithelial cells injury, cellular senescence, and chronic inflammatory stimulation (6). Although two clinical drugs were approved by Food and Drug Administration, none of them reverse the lung injury caused by disease or reduces the mortality of IPF (7). Consequently, patients with IPF have a poor clinical outcome, and the average survival time has been reported to be approximately 3~5 years from clinical symptoms onset (8). Owing to an ageing population, the incidence rate of IPF has been increasing (9). Although researchers have achieved encouraging results in the diagnosis and treatment of IPF recently, IPF patients remains incurable with remarkably mortality (10). Thus, it is crucial to identify reliable biomarkers of IPF for prognosis and targeted therapy.

Transcriptomics has been extensively used to study the potential mechanisms of disease occurrence and progression (11, 12). Significant insights into the complex pathological and biological mechanisms of IPF, including the role of matrix metalloproteases in IPF, have been reported (13, 14). Furthermore, research has been conducted for screening differentially expressed genes in IPF lung, which provided important mechanistic insights (15). Importantly, FKBP10, RXFP1, and PTPN11 were identified as novel regulators of IPF (16–18). Single-cell RNA sequencing revealed that senescence-associated genes and WNT signaling play crucial roles in mediating IPF progression (19). Several studies in recent years have reported potential prognostic biomarkers and explored the disease-associated biological pathways using public databases (20). However, no effective biomarkers have been identified so far for evaluating the prognosis of IPF patients and providing subsequent guidance for treatment selection.

Currently, lung biopsy is considered to be an ideal method for identifying molecular markers for the diagnosis and prognosis of IPF. However, its use is limited owing to the invasive nature of the procedure, with less than 30% of patients receive a biopsy, so its utility is limited. Several studies have reported that bronchoalveolar lavage (BAL) fluid (BALF), which is often used in the diagnosis of lung diseases, can be used to evaluate the stages of inflammatory response in interstitial lung disease (21, 22). In this study, the mRNA microarray datasets of BAL cells from Gene Expression Omnibus (GEO; http://www.ncbi.nlm.nih.gov/geo/) was obtained. The various hallmarks of IPF as risk factors for overall survival were analyzed and combined multiple bioinformatics methods were used to screen for biomarkers and develop a robust gene signature for the prognosis of IPF. Additionally, the prognostic value of this gene signature was verified using an independent external dataset. This study provided a novel perspective to understand the mechanisms of IPF and presents a risk model for predicting the overall survival of patients with IPF.

## Materials and Methods

### Data Acquisition and Processing

A total of 196 patients with IPF, including their clinical information, and 20 healthy donors were included in this study. The gene expression of BAL cells (GSE70866) was obtained from Gene Expression Omnibus (GEO) database (23). Among them, RNA microarray chips from 20 healthy donors and 112 patients with IPF (from Freiburg and Siena) were performed using Agilent-028004 SurePrint G3 Human GE 8x60K Microarray, while RNA microarray chips from 64 IPF patients (from Leuven) were evaluated by Agilent-039494 SurePrint G3 Human GE v2 8x60K Microarray. The basic information of patients was shown in **Table 1**. In addition, the “limma” and “sva” functions were performed to background-correct, normalize and remove batch effects from raw expression data (24, 25). The cohort of 112 patients with IPF was employed as the training set, and the cohort of 64 patients with IPF was used as a validation set. Ethical approval was not required in this study since all data was available in the public domain.

**Table 1:** The basic information of IPF patients.

Characteristic		Training set	Validation set
Status	Alive	36 (32.14%)	40 (62.50%)
	Dead	76 (67.86%)	24 (37.50%)
Age	<=65	40 (35.71%)	25 (39.06%)
	>65	72 (64.29%)	39 (60.94%)
Sex	Female	19 (16.96%)	13 (20.31%)
	Male	93 (83.04%)	51 (79.69%)

### Identification of Candidate Hallmarks

The “limma” package was applied to screen differentially expressed genes (p value<0.05). The gene sets of hallmarks were obtained from the Molecular Signatures Database (MSigDB), and the Z-score of hallmarks was quantified using a single-sample gene set enrichment analysis (ssGSEA) algorithm (R package ‘gsva’) based on transcriptome profiling data. Univariate Cox proportional-hazards (Cox-PH) regression was applied to estimate the significance of different hallmarks in IPF through the “survival” function.

### Weighted Gene Co-Expression Network Analysis

Weighted Gene Co-expression Network Analysis (WGCNA) was carried to identify the module that was most correlated with hypoxia based on transcriptome profiling data and ssGSEA scores. Firstly, the gene expression matrix was transformed into a similarity matrix by using the Pearson test between pairwise genes. Secondly, the similarity matrix was transformed to an adjacency matrix by Topological overlap measure (TOM). Gene co‐expression networks were generated by the WGCNA package. Module membership (MM) represented the correlation between module eigengenes and gene expression profiles. 369 candidate genes were identified with a threshold of correlation coefficient of MM >0.5 and the p-value <0.001.

### Construction of Risk Model

The univariate Cox regression was performed to assess the prognostic value of candidate genes. Then, the importance of the survival-related differential genes was calculated and ranked using the random forest *via* the “randomForestSRC” package. A risk model was built by multivariate Cox regression, and the risk score was calculated as follows: Risk Score = β1x1 +β2x2+β3x3+…+ βnxn.

### Correlation Analysis of Immune Cells

Single-Sample GSEA (ssGSEA) and MCP counter algorithm were applied to estimate the infiltration levels of immune cells based on specific gene expression signatures of immune cells. Subsequently, we explored the correlation between risk score and immune cells. Furthermore, we also analyzed the relationship between risk score and NLRP3.

### Additional Bioinformatic and Statistical Analyses

R software (version 3.6.3, http://www.r-project.org) was used to analyze data and plot graphs. Kaplan-Meier (K-M) curve plotted the relationship between score and clinical outcome, and the log-rank test was used to evaluate differences using “survival” and “survminer” packages. Univariate and multivariate Cox regressions were performed with the “survival” package. Subsequently, the Wilcox test was used to calculate the differences between the groups, and p<0.05 was considered significant. WGCNA was used to describe the correlation patterns among gene by following the protocol of WGCNA package. Time-dependent concordance index (C-index) and the receiver operating characteristic (ROC) analysis were applied to assess the predictive capacity of survival among different variables using R packages’ survConcordance’ and ‘survivalROC’. Then, gene set enrichment analysis (GSEA) was performed using GSEA software (http://software.broadinstitute.org/gsea). Finally, the calibration of the nomogram was plotted by the “rms” package.

## Results

### Identification of Risk Factors for Overall Survival in IPF

**Figure 1:**
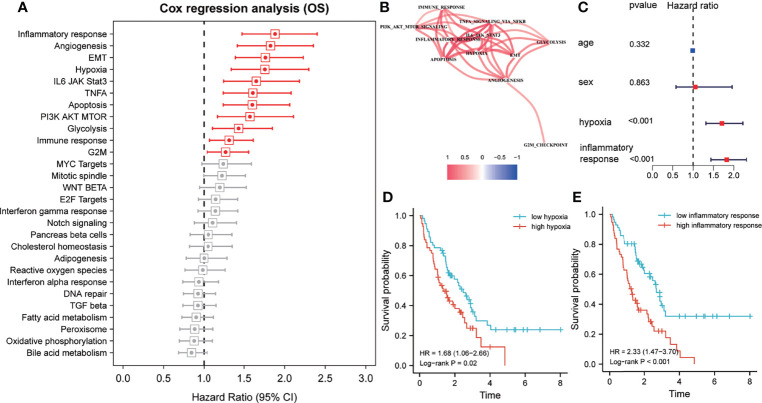
Inflammatory response and hypoxia are dominant risk factors for patients with idiopathic pulmonary fibrosis (IPF) in the training cohort. **(A)** Univariate Cox regression analysis for the prognostic value of various hallmarks. Red, p < 0.05; grey, p > 0.05. **(B)** Spearman correlation analysis was performed to construct a correlation network for prognosis-related hallmarks. **(C)** Multivariate Cox regression analyses of clinical factors and the inflammatory response and hypoxia-related signature. **(D)** Kaplan–Meier survival analysis of the single-sample gene set enrichment analysis (ssGSEA) score of hypoxias for overall survival. **(E)** Kaplan–Meier survival analysis of the ssGSEA score of inflammatory response for overall survival.

The z-scores of various hallmarks were calculated based on the transcriptome profiling of the training set and gene sets of MSigDB using ssGSEA. In addition, the hazard ratios (HR) of various hallmarks were calculated and ranked based on the survival information. The results indicated that the inflammatory response, angiogenesis, epithelial-mesenchymal transition, hypoxia, immune response, and other signaling pathways impacted the overall survival of patients with IPF from the training cohort (**Figure 1A**). Among these hallmarks, inflammatory response and hypoxia exhibited more powerful effects on survival compared to others. Then, a co-expression network of the survival-related hallmarks was generated based on their ssGSEA z-scores. The result revealed that compared to other hallmarks, inflammatory response and hypoxia showed a higher correlation (**Figure 1B**). Additionally, multivariate Cox analysis demonstrated that both inflammatory response and hypoxia were prognostic factors of IPF independent from other clinical features (including age and sex) (**Figure 1C**). Based on the median value of ssGSEA score of inflammatory response and hypoxia, the training cohort was divided into the high- and low-score groups, with inflammatory response and hypoxia presenting worse clinical outcomes as indicated by their high z-score (**Figures 1D, E**).

### Construction of Inflammatory Response and Hypoxia-Related Gene Signature

**Figure 2:**
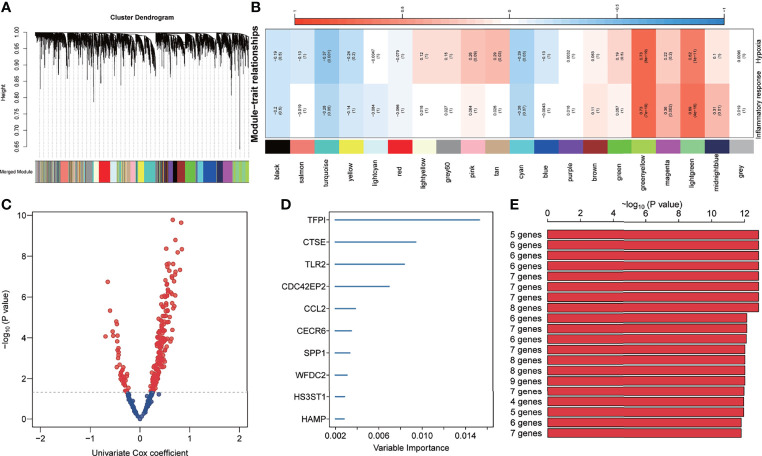
Development of inflammation response and hypoxia-related gene signature. **(A)** Cluster dendrogram and co-expression modules were identified using Weighted Gene Co-expression Network Analysis. Each colour represents one module. **(B)** Correlation analysis between the gene module and inflammatory response and hypoxia-related gene signature. **(C)** The prognosis-related genes in the green-yellow and light-green modules were screened using univariate Cox regression. **(D)** The top 10 genes were identified using random survival forest algorithms. **(E)** The top 20 gene signatures were ranked by -log10 p-value on a Kaplan–Meier plotter.

In total, 4072 genes were identified as differentially expressed genes between IPF and healthy donors. Then, WGCNA was used to construct a scale-free co-expression network based on the average linkage method, and 20 modules were generated (**Figure 2A**). Among them, both green-yellow and light green modules showed a strong positive correlation with inflammatory response and hypoxia (r>0.6, p<0.0001) (**Figure 2B**). Subsequently, univariate Cox regression was performed to examine the prognostic value of the 369 candidate genes from the green-yellow and light green modules, which revealed 42 and 174 genes to be negatively and positively associated with prognosis, respectively (**Figure 2C**). Furthermore, the random forest analysis was applied to rank and screen relative important genes based on their impact on overall survival. Consequently, TFPI, CTSE, TLR2, CDC42EP2, CCL2, CECR6, SPP1, WFDC2, HS3ST1, and HAMP were identified as the top 10 genes (**Figure 2D**). Approximately 1023 risk models were calculated for the different combinations of the 10 genes and K-M analysis was performed to screen for the best risk model. The Log-rank p-value of all risk models was calculated and ranked, and the top 20 was selected for further study (**Figure 2E**). The five-gene signature was identified as the final risk model, as it exhibited the smallest p-value and fewer risk genes, with the risk score = 0.44× HS3ST1+ 0.27× WFDC2 + 0.16 × SPP1 + 1.12 × TFPI + 1.06 × CDC42EP2.

### Five-Gene Signature Serves as a Risk Factor for Patients With IPF

**Figure 3:**
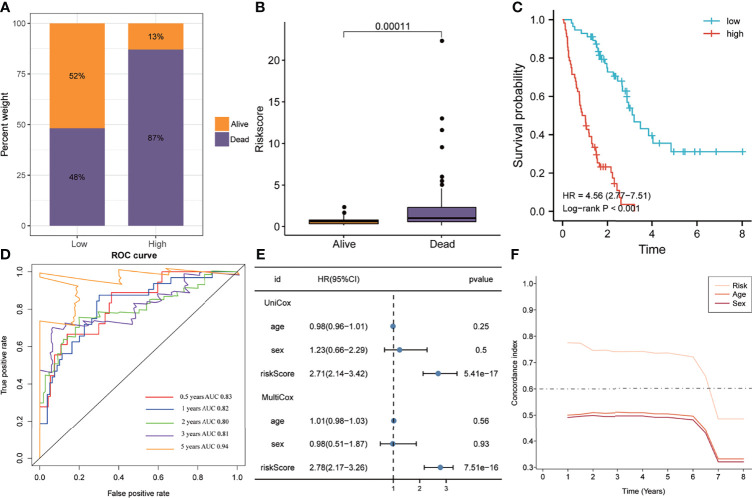
The predictive ability of the signature in the training cohort. **(A)** Distribution of survival status in the high- and low-risk groups of the training cohort. **(B)** The distribution of the gene signature risk scores between the patients who have died and patients who are alive. **(C)** Kaplan-Meier method was performed to plot the survival curves based on risk scores, which were compared using log-rank test. **(D)** The area under the receiver operating characteristic curve value of the risk model for 0.5-, 1-, 2-, 3- and 5-years is 0.83, 0.82, 0.80, 0.81 and 0.94 respectively. **(E)** Univariate and multivariate Cox regression analyses suggested that this risk model was an independent prognostic factor. **(F)** Time-dependent concordance index exhibits the excellent predictive ability of the model.

In the training set, the 112 patients were divided into high and low-risk groups based on the median of risk scores. The Bar plots demonstrated that the proportion of deaths in the high-risk group was 87%, significantly higher than the low-risk group (**Figure 3A**). Meanwhile, the risk score was significantly higher in patients who died during follow-up compared to survivors (**Figure 3B**). Furthermore, the K-M analysis indicated that the high-risk group had worse clinical outcomes than the low-risk group (HR=4.56, P<0.001, **Figure 3C**). Area under the ROC curve (AUC) analysis revealed the reliable predictive ability of the model for the overall survival of IPF, with the 0.5-, 1-, 2-, 3-, and 5-years AUC values of 0.83, 0.82, 0.80, 0.81, and 0.94, respectively (**Figure 3D**). Furthermore, univariate and multivariate Cox analyses indicated that the five-gene signature was an independent risk factor (HR=2.78, P<0.0001, **Figure 3E**). In addition, the C-index of the five-gene signature was significantly higher than that of age and sex, and was approximately 0.8 for 1-5 years (**Figure 3F**).

### Validation of Five-Gene Signature

**Figure 4:**
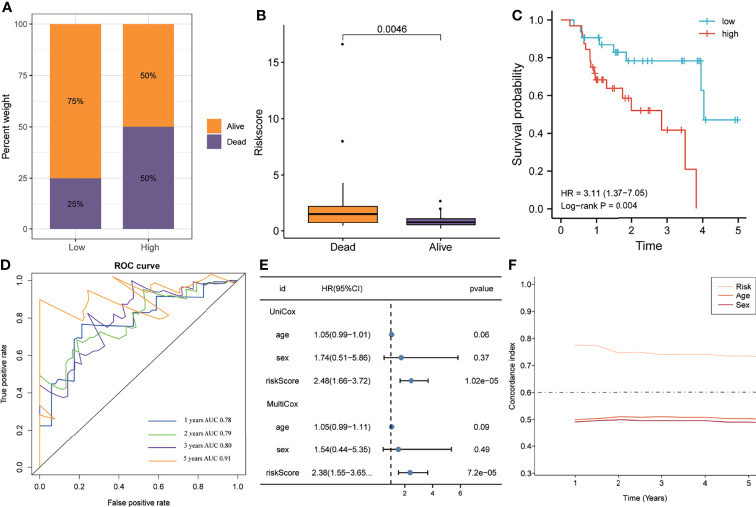
The predictive ability of the gene signature in the validation cohort. **(A)** The distribution of survival status in the high- and low-risk groups. **(B)** The distribution of the gene signature risk scores between patients who have died and patients who are alive. **(C)** Survival curves were plotted using the Kaplan–Meier method and compared using the log-rank test. **(D)** The area under the receiver operating characteristic curve value of the risk model for 1-, 2-, 3- and 5-years is 0.78, 0.79, 0.80 and 0.91 respectively. **(E)** Univariate and multivariate Cox regression analyses of the risk model. **(F)** Time-dependent concordance index exhibits the excellent predictive ability of the risk model.

Similar to the training cohort, the high-risk group in the validation cohort had a higher mortality rate than the low-risk group, with a significant elevation of the risk score in patients who died (**Figures 4A, B**). The K-M plotter showed that patients with IPF in the high-risk group exhibited poorer overall survival than the low-risk group (HR=3.11, P=0.004, **Figure 4C**). The ROC analyses indicated that the five-gene signature had a good prediction accuracy, with the AUC for 1-, 2-, 3-, and 5-years being 0.78, 0.79, 0.80, and 0.91, respectively (**Figure 4D**). The multivariate Cox regression analysis demonstrated that the five-gene signature served as an independent prognostic factor (HR=2.38, P<0.0001, **Figure 4E**), which was further verified by the C-index that showed that the five-gene signature to have excellent prediction value in the validation cohort (**Figure 4F**).

### Association of Inflammatory Response and Hypoxia With Risk Score

**Figure 5:**
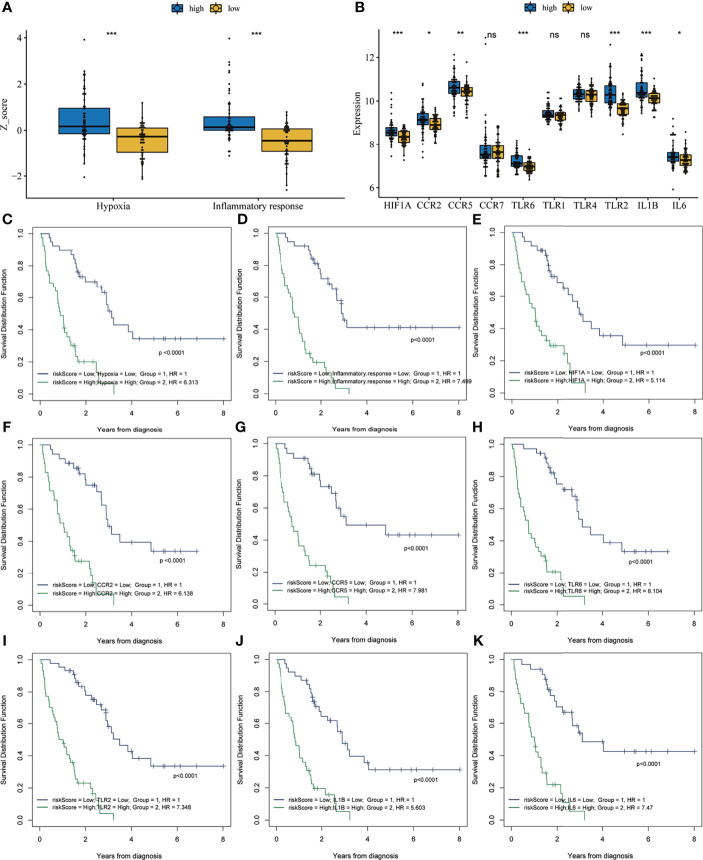
Correlation analysis between risk score and inflammatory response and hypoxia. **(A)** The single-sample gene set enrichment analysis score of hypoxia and inflammatory response in the high- and low-risk groups. **(B)** The expression of inflammation response and hypoxia-related genes in the high- and low-risk groups. Survival analysis of the combination of risk score with hypoxia **(C)**, inflammatory response **(D)**, HIF1A **(E)**, CCR2 **(F)**, CCR5 **(G)**, TLR6 **(H)**, TLR2 **(I)**, IL1B **(J)** and IL6 **(K)**. *P < 0.05, **P < 0.01, ***P < 0.001, NS, no significance.

**Figure 6:**
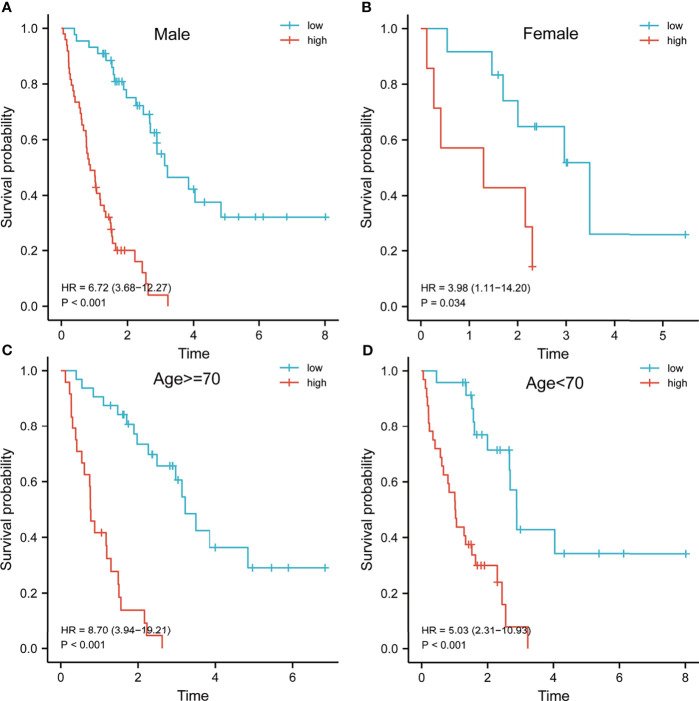
Survival analysis of patients with idiopathic pulmonary fibrosis in different subgroups. Kaplan–Meier survival analysis of the risk model in different subgroups, including male **(A)**, female **(B)**, age > = 70 years **(C)** and age < 70 years **(D)**.

**Figure 7:**
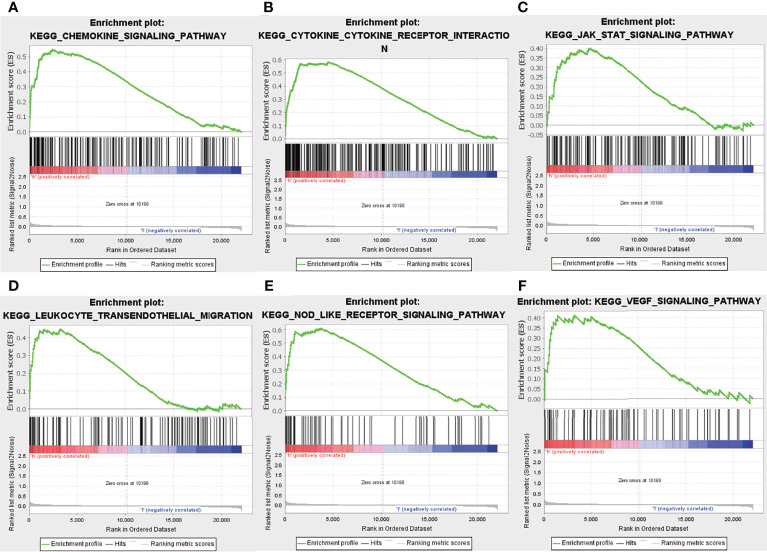
Gene set enrichment analysis. Enrichment pathway between the high- and low-risk groups, including chemokine signalling pathway **(A)**, cytokine receptor interaction **(B)**, JAK-STAT signalling pathway **(C)**, leukocyte transendothelial migration **(D)**, Nod-like receptor signalling pathway **(E)** and VEGF signalling pathway **(F)**.

Previous results have suggested that the ssGSEA score of inflammatory response and hypoxia was associated with the clinical outcome of patients with IPF in the training set. Following this, the analysis of the relationship between inflammatory response and hypoxia with the five-gene signature, demonstrated that the high-risk group had a higher ssGSEA score of inflammatory response and hypoxia than that of the low-risk group (**Figure 5A**). Meanwhile, the expression of inflammatory response and hypoxia-related genes, including HIF1A, CCR2, CCR5, TLR6, TLR2, IL1B, and IL6, was higher in the high-risk group than low-risk group (**Figure 5B**). In order to further explore the potential relationship of inflammatory response, hypoxia and related genes with the risk score, the correlation of risk score associated inflammatory response and hypoxia and related genes with clinical performance was analyzed, which showed that the patients with IPF having a high-risk score and high ssGSEA scores of hypoxias or inflammatory response presented worse clinical outcomes (**Figures 5C, D**). In addition, the patients with IPF showing a high expression of related-genes, including HIF1A, CCR2, CCR5, TLR2, IL1B, and IL6, and high risk-scores had a poorer prognosis (**Figures 5E–K**). Subsequent investigation of the five-gene signature on clinical outcome in different subgroups revealed that patients with IPF in the high-risk group had a poor prognosis in both male and female patients (**Figures 6A, B**). In both subgroups of patients ages ≥ 70 years and those aged < 70 years, the risk model retained its prognostic capacity to discriminate high-risk subset (**Figures 6C, D**). The GSEA analyses, which investigated the significantly altered KEGG pathway between the high- and low-risk groups, demonstrated that the chemokine signalling pathway, cytokine receptor interaction, JAK-STAT signalling pathway, leukocyte transendothelial migration, NOD-like receptor signalling pathway and VEGF signalling pathway were enriched in the high-risk group (**Figures 7A–F**). This further confirmed that the high-risk group was association with inflammation response and hypoxia status.

### Comprehensive Analysis of Immune Cells Between High- and Low-Risk Groups

**Figure 8:**
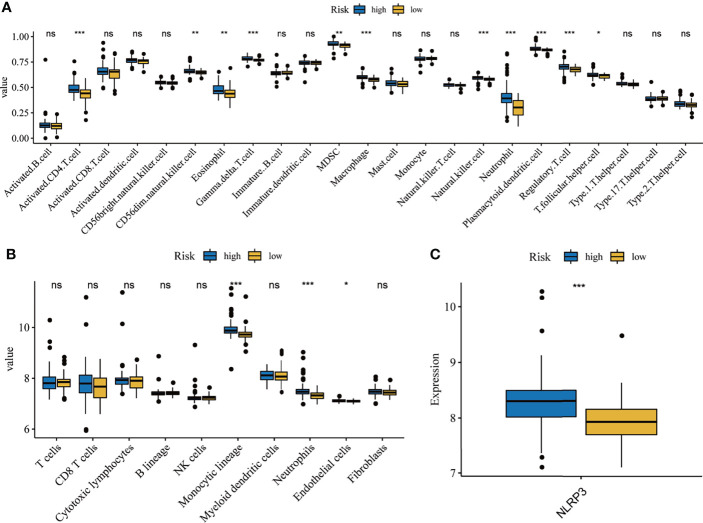
Correlation analysis of immune and inflammation. Differentially composition of infiltrated immune cells between high- and low-risk group *via* ssGSEA **(A)** and MCP counter **(B)**analysis. **(C)** The expression levels of NLRP3 between high-and low-risk groups. *p < 0.05, **p < 0.01, ***p < 0.001, ns, no significance.

Although previous results indicated that the hallmarks of hypoxia and inflammatory response and five-gene signature heavily influences the prognosis of IPF patients, its potential function warranted additional explored. As reported, the BAL contains a large number of white blood cells, and they play crucial roles in the development and deterioration of organ fibrosis (26, 27). It is unclear whether there are differences in immune cell infiltration and inflammatory factors between high- and low- risk groups. Here, we analyzed the correlation between the infiltration level of immune cell and risk score, based on ssGSEA and MCP counter algorithm. Then, the results from ssGSEA analysis demonstrated that the high-risk group exhibited higher immune cell infiltration, including CD4+ T cell, natural killer cell, eosinophil, MDSC, Macrophage, neutrophil, dendritic cell, regulatory T cell, and follicular helper cell, compared to low-risk group (**Figure 8A**). Meanwhile, the results from MCP counter indicated that monocytic lineage and neutrophils abundance in the high-risk group was significantly higher that the low-risk group (**Figure 8B**). Furthermore, we also found that high-risk group had higher NLRP3 expression, which is known NLRP3 inflammasome drive chronic inflammation, compared to low-risk group (**Figure 8C**).

### Construction of a Nomogram

**Figure 9:**
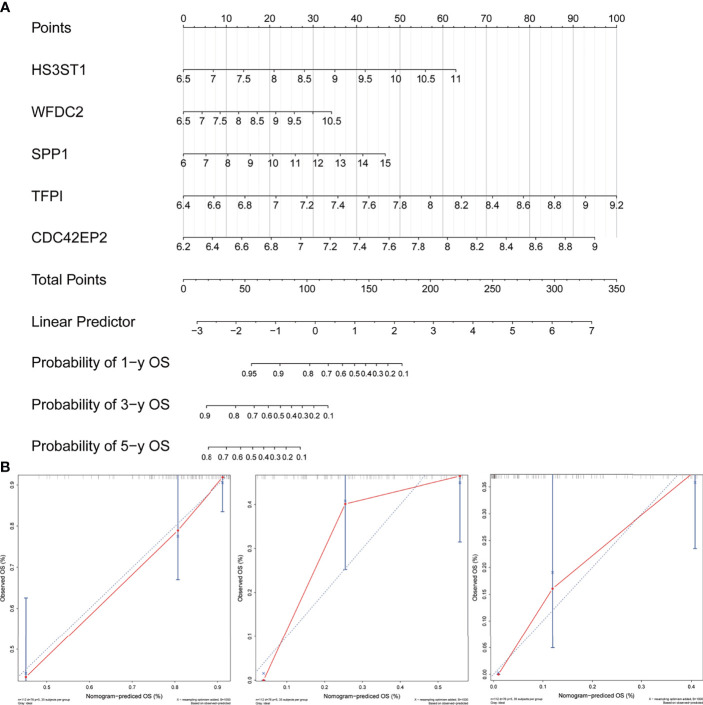
Development and evaluation of the nomogram. **(A)** A combination of five genes’ expression was used to construct a nomogram for predicting the 1-, 3- and 5-year overall survival. **(B)** Calibration curves demonstrate that the nomogram-predicted overall survival probabilities correspond closely to the observed probabilities for 1-, 3- and 5-years in patients with idiopathic pulmonary fibrosis.

In order to quantify the risk assessment of individual patients with IPF and enhance the clinical applicability, a nomogram with five genes was constructed to predict the probability of 1-, 3-, and 5-years overall survival (**Figure 9A**). Calibration plots assessing the performance of the nomogram demonstrated its excellent prediction accuracy for 1-, 3- and 5-year survival probability (45° line, **Figure 9B**).

## Discussion

Early-stage symptoms of IPF involve the lung tissue showing a strong inflammatory response, including large inflammatory cell infiltration, macrophage activation and inflammatory mediator release, which damages the capillary endothelial cells and alveolar epithelial cells (28). Furthermore, macrophages are activated along with the neutrophils and monocytes being recruited when the injury persists, producing more inflammatory factors and reactive oxygen species, which further aggravates the epithelial cell injury (29). Hypoxia, a prominent characteristic of IPF, has been reported to activate multiple pathways in IPF, including HIF1A and VEGF pathways, which can promote fibroblast proliferation and lead to the IPF process deterioration (30, 31). Nintedanib and pirfenidone are approved medications for the treatment of IPF, and they inhibit the proliferation of vascular cells and anti-inflammation respectively (2, 32–34). Therefore, inflammation and hypoxia play an important role in the pathogenesis and prognosis of IPF. However, the role of inflammation and hypoxia in IPF remains to be completely elucidated.

This study identified the inflammation response and hypoxia as risk factors for overall survival in patients with IPF from different hallmarks using ssGSEA and Cox regression models. As inflammation response and hypoxia are complex regulatory processes involving multiple genes (35–37). Therefore, we used the WGCNA to identify the inflammation response and hypoxia-related expression module. Then, univariate, random forest, and multivariate Cox regression were performed to screen the relative importance of genes and build an inflammation response and hypoxia-related signature. At the same time, the K-M analyses and ROC were employed to assess the prediction accuracy. Subsequently, we verified the reliability of the risk model in an independent external dataset. These results indicated that the developed risk model could directly distinguish the high-risk patients from low-risk patients, and high-risk patients presented worse overall survival in both training and validation cohorts. As the risk model successfully used the inflammation response and hypoxia network, it provides a novel perspective for risk assessment before nintedanib and pirfenidone treatment in patients with IPF.

GSEA analysis suggested that the pathways of immune and inflammation response were enriched in the high-risk group. Meanwhile, a deeper analysis of immune correlates also confirmed that the high-risk group had more extensive infiltrations of inflammatory cells compared to low-risk group. These results are consistent with that inflammatory response promote fibrosis process of IPF patients (38). The NLRP3 inflammasome play a crucial role in regulation of inflammatory response (39). Our result also revealed that high-risk group had higher expression levels of NLRP3 than low-risk group.

The application of prognostic markers in IPF is lacking despite several studies reporting that clinical, radiological and demographic factors can be used to construct mortality risk models for patients with IPF (40, 41). In this study, five genes (HS3ST1, WFDC2, SPP1, TFPI, and CDC42EP2) were identified as important prognostic biomarkers. HS3ST1, heparan sulphate-glucosamine 3-sulfotransferase 1, is a member of the heparan sulphate biosynthetic enzyme family, which is involved in the anti-inflammatory activity of diverse diseases (42, 43). Additionally, HS3ST1 has been reported to play a major role in the development of colorectal cancer. Moreover, HS3ST1 and PD1 could be promising antigen-specific immunotherapy targets for colorectal cancer (44). Notably, the overexpression of HS3ST1 was associated with poor prognosis while acting as a hypoxia-related biomarker in bladder cancer (45, 46). Previous studies suggest that HS3ST1 was significantly correlated with inflammation and hypoxia, which is consistent with our results and thereby indicates that HS3ST1 could be a promising target for IPF. WFDC2, also called HE4, was identified as a prognostic biomarker for various cancer types and is involved in the regulation of multiple pathways, including EGFR and STAT3 (47–49). A recent clinical study shows that aberrant HE4 expression of patients with IPF was associated with a poor clinical outcome; however, this aspect requires further investigation (50). WFDC2 was also identified as a prognostic biomarker based on the transcriptome profile of BALF, providing new insights into understanding the role of WFDC2 in IPF development. Pardo et al. reported that SPP1 was overexpressed in IPF alveolar epithelial cells and significantly enhanced in BALF, indicating its potential as a therapeutic target for IPF (51). Christina Morse et al. found that SPP1 expression was significantly increased in IPF macrophages by single-cell transcriptomes analyses and indicated that macrophages with high SPP1 expression further promote epithelial fibrosis and enhance inflammation response (52). These are in agreement with our findings, suggesting that SPP1 could be an ideal prognostic biomarker for IPF. Another study has shown that TFPI levels were remarkedly increased in BALF of IPF patients (53). CDC42EP2 was reported to be involved in the formation of the organization of the actin cytoskeleton (54). However, the role of CDC42EP2 in IPF has not been explored. Thus, the biological functions associated with inflammation response and hypoxia of the five-gene signature in IPF still require further exploration.

Since our research was a retrospective cohort study, there were inevitably some limitations. Firstly, although the training and validation sets were included, validation using more clinical datasets is required. Secondly, microarray panel has a limitation to identify novel gene models. Thirdly, the potential inflammation response and hypoxia-related biological role of the five-gene signature requires further *in vivo* and *in vitro* verifications.

## Conclusion

This study reports the construction of a novel inflammation- and hypoxia-related five-gene signature for patients with IPF based on BAL cells. Based on the expression profiles of these five genes, a nomogram was constructed to quantify risk assessment for individual patients. This gene signature could prove beneficial to the treatment monitoring and follow-up management of patients with IPF.

## Data Availability Statement

The original contributions presented in the study are included in the article/supplementary material. Further inquiries can be directed to the corresponding author.

## Author Contributions

WL and JL designed the study and revised the manuscript. JL and LG collected, analyzed, and interpreted the data. JL drafted the manuscript. All authors have read and approved the final manuscript.

## Conflict of Interest

The authors declare that the research was conducted in the absence of any commercial or financial relationships that could be construed as a potential conflict of interest.

## Publisher’s Note

All claims expressed in this article are solely those of the authors and do not necessarily represent those of their affiliated organizations, or those of the publisher, the editors and the reviewers. Any product that may be evaluated in this article, or claim that may be made by its manufacturer, is not guaranteed or endorsed by the publisher.
